# Special Issue: “The Complexity of the Potyviral Interaction Network”

**DOI:** 10.3390/v12080874

**Published:** 2020-08-11

**Authors:** Sylvie German-Retana, Kristiina Mäkinen

**Affiliations:** 1 UMR 1332 Biologie du Fruit et Pathologie, INRAE, Univ. Bordeaux, 71 Av. E. Bourlaux, CS 20032, 33882 Villenave d’Ornon Cedex, France; 2 Department of Microbiology and Viikki Plant Science Centre, University of Helsinki, 00014 Helsinki, Finland

Many potyvirus species are among the most economically-significant plant viruses as they cause substantial yield losses to crop plants globally. Investigating their infectious cycles and host–potyvirus interactions is therefore essential for the development of anti-viral strategies and resistant cultivars. The thirteen original articles included in the Special Issue on “The Complexity of the Potyviral Interaction Network” shed light on the current state of potyvirus research in three main areas: (i) complex interactions between multifunctional potyviral proteins and antiviral or proviral host factors; (ii) molecular and physiological responses of the plant to potyvirus infection; and (iii) potyvirus evolution and phylogeny.

The review of Shen et al. [1] illustrates how the RNA-dependent RNA polymerase, as a multifunctional potyviral protein, can recruit pro-viral host factors crucial for the infection cycle or for the suppression of immunity defense, while being targeted by antiviral responses such as autophagy and effector-triggered immunity. The two complementary studies of Ala-Poikela et al. [2] and Saha and Mäkinen [3] provide more insights into the role of translation initiation factors in potyvirus infection. Potyviral proteins VPg and HCpro and host proteins eIF4E and eIF(iso)4E form an interaction network, which likely contributes to RNAi suppression [2]. The binding of eIF(iso)4E to VPg is proposed to mediate RNA stabilization, transfer to the RNA silencing suppression pathway, and further to polysomes for viral protein synthesis [3]. The study of Hervás et al. [4] illustrates how post-translational modifications of potyviral capsid protein through O-GlcNAcylation and phosphorylation in herbaceous and ligneous hosts may contribute to both general and strain-specific strategies deployed during plant–virus interactions. The study of Sabharwal and Savithri highlights the role of intrinsically disordered domains in the multiple functions of potyviral proteins and describes how potyvirus-like particles can be used as nanocarriers for biomedical applications [5].

In an additional four articles, the molecular responses of crops to potyvirus infection are analyzed through high-throughput transcriptomics approaches. Different pathosystems that yield different outcomes in potyviral infection are analyzed, which include antagonism or synergism induced in papaya by mixed-infection of papaya ringspot potyvirus and papaya mosaic potexvirus [6], tomato recovery from potato virus Y (PVY) infection [7], potato susceptibility or resistance to PVY [8], soybean susceptibility to soybean mosaic virus depending on light conditions [9], and transition from mild to severe tobacco etch disease in tobacco [10]. In particular, these studies highlight how the outcome of an infection can be linked to the regulation of genes involved in plant immunity and the role played by small RNAs in those processes. In their original paper, Tarazona et al. [10] applied Dynamical Network Biomarker (DNB) theory for the first time to a plant virus to identify genes or network elements that could be used as early-warnings of the transition from health to disease. Through physiological and ultrastructural analyses, Otulak-Koziel et al. [11] highlighted the role of plant expansins and extensins in the dynamics of the cell wall structure during incompatible interactions in resistant and compatible interactions in susceptible potato upon potato virus Y infections.

Finally, two complementary articles on potyvirus phylogeny complete the contributions to this Special Issue. The study of Moury and Desbiez [12] describe how potyviruses may have undergone numerous host range changes in a relatively short amount of time and that crop plants have contributed substantially to their diversification [12]. The paper of Gibbs et al. [13] provides a thorough review of potyvirus evolution, starting from a historical perspective and progressing to the taxonomical relationships between potyviruses and their temporal and geographical distribution.

Collectively, the papers in this Special Issue exemplify the diversity of studies focused on this major plant virus group. We wish to express our sincere thanks to all authors who contributed to the Special Issue “The Complexity of the Potyviral Interaction Network” and our gratitude to the reviewers who vetted the contributions.
